# Research progress of CD73-adenosine signaling regulating hepatocellular carcinoma through tumor microenvironment

**DOI:** 10.1186/s13046-025-03416-5

**Published:** 2025-05-26

**Authors:** Liang Shan, Mingxu Gong, Dandan Zhai, Xiangyun Meng, Jianjun Liu, Xiongwen Lv

**Affiliations:** 1 Department of Pharmacy, The Second People’s Hospital of Hefei (Hefei Hospital Affiliated to Anhui Medical University), Hefei, Anhui 230000 China; 2 Anhui Province Key Laboratory of Major Autoimmune Diseases, Anhui Medical University, Hefei, Anhui 230032 China; 3 Inflammation and Immune Mediated Diseases Laboratory of Anhui Province, Hefei, Anhui 230032 China; 4 The Key Laboratory of Major Autoimmune Diseases, Hefei, Anhui Province 230032 China

**Keywords:** CD73-adenosine signaling pathway, Hepatocellular carcinoma, Tumor microenvironment, Adenosine, Adenosine receptor

## Abstract

Adenosine signaling pathway is a kind of signal regulation hub widely existing in human body, which is involved in a series of physiological processes such as energy supply of body cells. CD73 is a highly concerned signaling protein in purine adenosine pathway, and its role in tumor development and prognosis has been paid more and more attention in recent years, especially in hepatocellular carcinoma (HCC). In this paper, the specific mechanism by which CD73-adenosine signaling regulates tumor microenvironment (TME) of liver cancer tumors was analyzed in detail, highlighting the importance of this pathway as a therapeutic target to combat tumor immunosuppression and enhance the anti-tumor immune response to prevent and treat hepatocellular carcinoma (HCC). In addition, a variety of current targeted therapeutic strategies for adenosine metabolic pathways are summarized, including the development of new drugs in the stage of preclinical research and clinical trials, and the mechanism of action, implementation possibility, and clinical effects of these therapies are discussed. By summarizing the latest scientific research results, in this review, we attempt to paint a panorama of the mechanism of adenosine action in tumor immunotherapy, with the aim to provide a solid theoretical basis and practical guidance for subsequent research and clinical application, ultimately promoting the development of more accurate and efficient tumor immunotherapy.

## Background

**Fig. 1:**
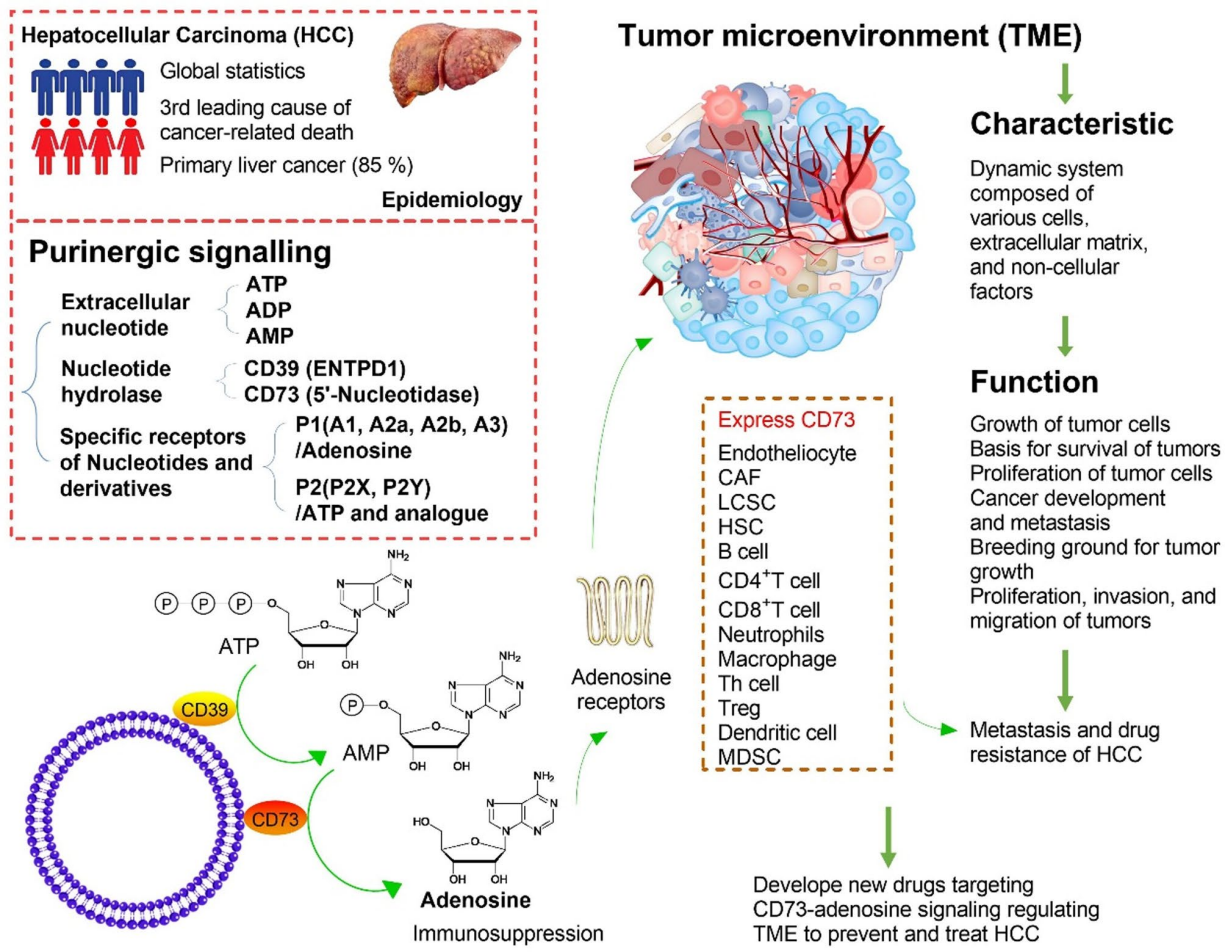
CD73 and adenosine receptors are highly expressed in the liver tissues of patients with hepatocellular carcinoma (HCC). CD73 and adenosine receptors are expressed in a variety of cells. The regulation of CD73-adenosine signals in the tumor microenvironment (TME) to inhibit HCC progression is a current research hotspot

Liver cancer, the third leading cause of cancer-related death worldwide, is a multi-factor–induced, multi-gene–involved and complex digestive system malignancy [1]. Hepatocellular carcinoma (HCC) accounts for more than 85% of primary liver cancers [2]. Immunoinflammatory microenvironment-driven tumor development and treatment are major challenges in the prevention and treatment of HCC [3]. It is of great scientific significance to deeply understand the regulatory mechanism of the tumor immune microenvironment and to discover and elucidate the characteristics and functional remodeling of immune cells in the immune microenvironment and the mechanism of HCC progression [4]. Purinergic signaling participates in the pathophysiological regulation of many diseases, including liver disease [5]. Purinergic signaling is chiefly composed of extracellular nucleotides, nucleotide hydrolases (e.g., CD39, ENTPD1, CD73), nucleotides, and derivatives of specific receptors [6]. The purine receptors are divided into two categories, P1 receptors acting on adenosine, and P2 receptors acting on adenosine triphosphate (ATP) and its analogues [7]. Four subtypes of P1 receptor, including A1R, A2aR, A2bR, and A3R, have been cloned. P2 receptors are divided into P2X and P2Y groups [8]. CD73 plays an inhibitory role in the tumor microenvironment (TME) by mediating adenosine production [9]. Adenosine can inhibit the function of tumor-killing immune cells, such as effector T cells, natural killer (NK) cells, and dendritic cells (DCs), and enhance the function of suppressive immune cells, including regulatory T cells, myeloid suppressor cells, and tumor-associated macrophages [10]. At present, many companies at home and abroad are developing new drugs targeting CD73-adenosine to regulate the TME to prevent and treat HCC [11]. CD73 is expressed in a variety of human tumors, especially in HCC cells, cancer-associated fibroblasts (CAFs), and endothelial cells, but also in myeloid cells, NK cells, and T cells [12]. Numerous studies have described a strong association between elevated CD73 levels and adverse clinical outcomes [13]. In this article, we review recent progress in the immunotherapy of HCC by targeting CD73-adenosine signaling to regulate the TME of HCC (Fig. 1).

## TME regulates the development and progression of HCC

**Fig. 2:**
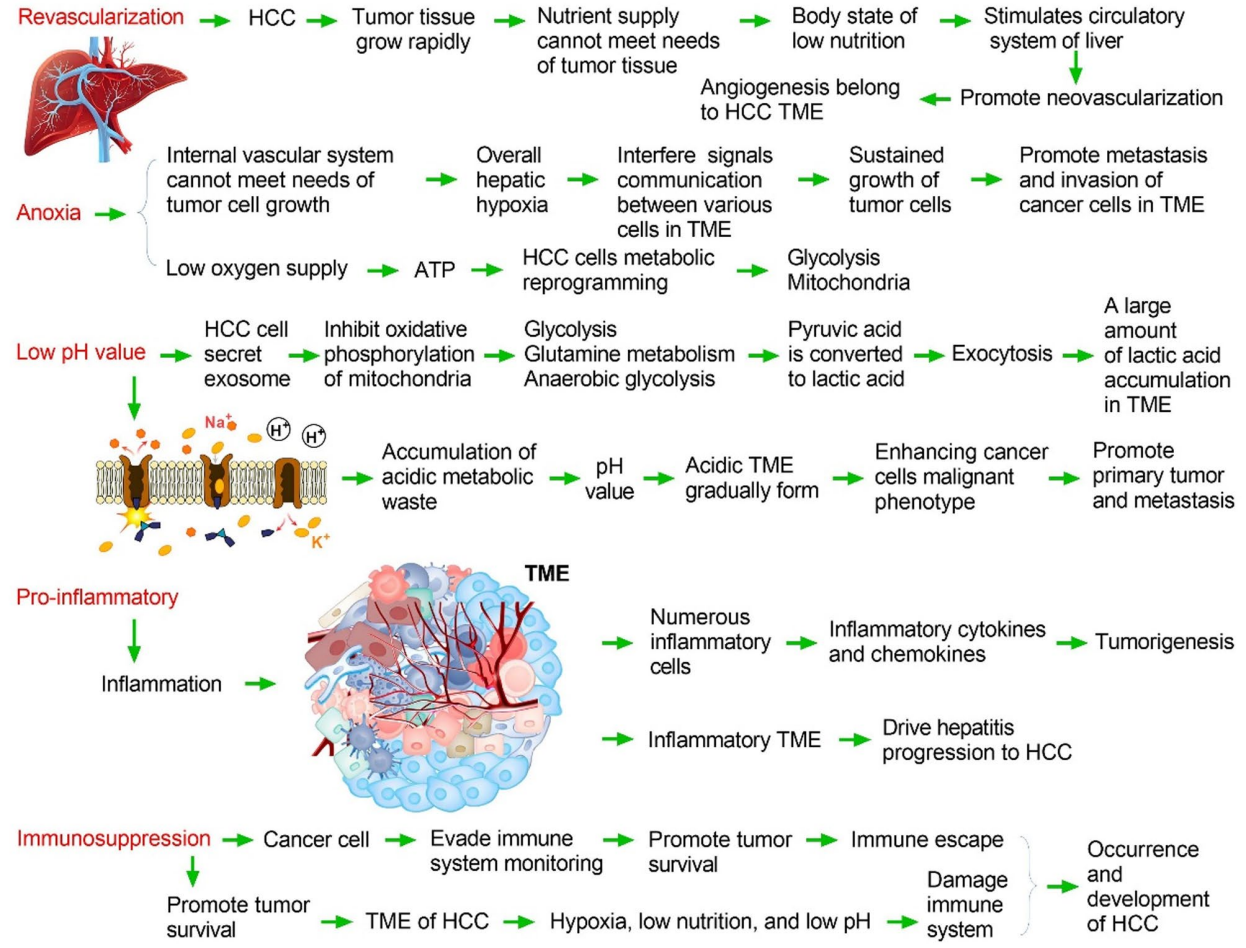
In the tumor microenvironment (TME) of hepatocellular carcinoma (HCC), the immune system is destroyed and rebuilt under an adverse environment, constituting hypoxia, low nutrition, low pH, and blood flow changes; this leads to the formation of a new systemic and local immune microenvironment that is suitable for tumor survival, regulating inflammation, and liver fibrosis, assisting tumors to escape immune system monitoring, leading to the occurrence and development of HCC

The TME refers to the local environment in which tumor cells reside [14]. It not only includes the tumor cells themselves, but also the surrounding support cells, extracellular matrix (ECM), blood vessels, immune cells, and a series of signaling molecules [15]. Most of the TME is occupied by the tumor, and it constitutes the mesenchyme of the tumor [16]. Low oxygen levels, high lactate levels, extracellular acidosis, and low nutrient content are notable features of the TME [17]. A variety of cells exist in the TME, including mesenchymal stem cells (MSCs), fibroblasts, endothelial cells, and immune cells. The TME can secrete cytokines and growth factors [18]. CAFs are among the most abundant cells in the TME, creating conditions for tumor growth and progression [19]. The interaction between the TME and the activation/inhibition signaling network may determine tumor progression [20]. Since 2017, other therapies to modulate the liver TME have emerged [21]. The liver immune microenvironment contains a large number of immune cells, including neutrophils, monocytes, resident macrophages (Kupffer cells [KCs]), NK cells, natural killer T (NKT) cells, and liver transport and/or resident lymphocytes (B, CD8 + T, CD4 + T, and γδ T cells) [22]. There is an overall balance between anti-inflammatory cytokines (interleukin [IL]-10, IL-13, and TGF-β) and pro-inflammatory cytokines (IL-2, IL-7, IL-12, IL-15, and interferon [IFN]-γ), maintaining homeostasis in vivo [23]. In recent years, purine signaling pathways have emerged as important players in cancer progression, with extracellular ATP, adenosine diphosphate (ADP), and adenosine being major signaling molecules [24]. The immunosuppressive metabolite adenosine is a component of the TME [25]. As the main enzyme that catalyzes adenosine production, CD73 is critical in inhibiting adequate anti-tumor immune responses, mainly through the production of adenosine, but also by promoting cancer cell proliferation, tumor growth, angiogenesis, and metastasis, warranting further study (Fig. 2).

### TME promotes vascular regeneration at the tumor site

HCC is a highly angiogenic cancer, and angiogenesis plays an important role in tumor growth, early metastasis, and poor survival [26]. The cell components of the TME system include hepatic stellate cells (HSCs), fibroblasts, immune cells, and endothelial cells [27]. Non-cellular components include growth factors (fibroblast growth factor [FGF], hepatocyte growth factor [HGF], and vascular endothelial growth factor [VEGF]), proteolytic enzymes, ECM proteins, and inflammatory factors [28]. The activated HSCs secrete angiogenic growth factor, which, together with VEGF, stimulate angiogenesis, form a new vascular system within the TME, and provide various nutrients for tumor growth [29, 30]. At present, many angiogenesis inhibitors have been approved for the clinical treatment of liver cancer [31]. Anti-VEGF-VEGFR drugs generally include the following categories: antibody drugs directly targeting VEGF and VEGFR proteins, such as bevacizumab; tyrosine kinase signaling pathway inhibitors, such as sorafenib; and types of fusion proteins and immunomodulators [32]. Combined with clinical findings, the clinical benefits of angiogenesis inhibitors are not obvious and have therapeutic limitations, which may be due to the regulation of angiogenesis networks involving multiple proteins or signaling pathways [33]. When a protein is inhibited by a single target drug, there is a compensatory phenomenon of angiogenesis, and the structure of new blood vessels in patients with tumors is incomplete and the permeability is poor; thus, some therapeutic drugs cannot effectively reach the lesion site (Fig. 2) [34].

### Hypoxia is an important characteristic of the HCC TME

Hypoxia plays an important role in tumor cell characteristics [35]. Studies have confirmed that hypoxia is closely related to the genesis, proliferation, apoptosis, autophagy, and metabolism of tumor cells [36]. Tumor cells are in a hypoxic environment for a long time [37]. Not only can tumor cells adapt to hypoxia, but hypoxia has also become a living environment for tumor cells to grow, proliferate, and maintain their characteristics [38]. Hypoxia not only controls cell proliferation but also apoptosis [39]. During the process from the beginning of malignant transformation to the formation of highly malignant tumors, malignant cells that can adapt to the hypoxic microenvironment survive and continue to proliferate to form highly malignant tumor tissues, whereas the cells in the inappropriate environment disappear through apoptosis and other mechanisms [40]. Most of the activities of tumor tissues are thought to be related to the anoxic microenvironment [41]. Hypoxia-inducible factors (HIFs) are some of the most important endogenous transcription factors produced under hypoxia, serving as the upstream factor responsible for causing many hypoxia reactions [42]. Among them, HIF-1 is the most widely distributed, most important, and most thoroughly studied, with HIF-1α as its functional monomer [43]. The expression of HIF-1α has been shown to be positively correlated with the severity of hypoxia, and the expression of HIF-1α is higher in regions with more severe hypoxia [44]. In addition, within a short time after the occurrence of hypoxia, HIF-1α in the cell is rapidly increased to a certain level [45]. The expression of CD73 is regulated by HIF-1, transforming growth factor (TGF)-β, EGFR, AKT, β-catenin, and other molecules, especially HIF-1, which plays the function of a transcription factor [46]. Hypoxia is an important feature of the TME [47]. Hypoxia induces HIF-1 upregulation, leading to the widespread expression of CD73 in the TME [48].

Current studies have confirmed that *HIF-1α* can directly regulate more than 60 target genes, and new target genes are constantly being discovered [49]. In tumor tissue, HIF-1α is closely related to tumor progression [50]. HIF-1α can regulate cell apoptosis and proliferation, enabling tumors to selectively multiply tumor clones that are more suitable for survival in anoxic microenvironments [51]. HIF-1α regulates a key enzyme involved in glycolysis, enabling tumor cells to survive hypoxia [52]. Anticancer therapies trigger the release of ATP from tumor cells, leading to the rapid formation of adenosine by the exonucleoenzymes CD39 and CD73, which subsequently aggravates immunosuppression in the TME [53]. ATP is the energy source of various cells, and several studies have found that downregulation of metabolic reprogramming in HCC cells can inhibit their growth [53, 54]. In addition, adenosine deaminase (ADA) can metabolize adenosine to inosine (INO), which causes severe metabolic reprogramming, reducing glycolysis and increasing mitochondrial and glycolytic capacity; however, the specific mechanism still needs to be further studied [55]. In conclusion, hypoxia is common in all solid tumors, and the HIF signaling pathway is involved in the inflammatory response, immunosuppression, and activation of a variety of cancer-promoting biological effects. HIF can lead to the aggregation of regulatory T cells (Tregs) and macrophages, promote sorafenib resistance, and is a potential biomarker for the diagnosis, prognosis, and recurrence of HCC (Fig. 2).

### TME of HCC exhibits a low pH (acidity)

Under normal circumstances, the pH levels inside and outside the cells of the human body are maintained within a certain range, which is essential for maintaining the physiological function of the human body [56]. The pH of the TME is usually between 6.5 and 7.5, slightly lower than the pH of normal cells [57]. The low pH of the TME of HCC may be related to the excessive production of lactic acid or abnormal glucose metabolism of tumor cells in an anoxic environment [58]. This is because tumor cells have certain metabolic characteristics and will produce vast amounts of lactic and carbonic acids, making the surrounding environment acidic and promoting the growth and metastasis of tumor cells [59]. The acidic condition of the TME affects cell survival and proliferation to some extent [60]. First, an acidic environment can reduce the stability of the ECM, facilitating the invasion of surrounding tissues by tumor cells [61]. Second, the acidic environment can also affect the metabolic activity of tumor cells, reducing their energy production and DNA repair ability, thereby increasing the susceptibility and mortality of cells [62]. An acidic environment can also affect the function of immune cells and inhibit the killing effect of the immune system on tumor cells [63]. Research on the pH range of the TME is still in its infancy and needs to be further explored. In future studies, pH changes in the TME can be monitored by employing non-invasive techniques such as magnetic resonance imaging and spectroscopy. In addition, the metabolic characteristics and gene expression profiles of tumor cells can be combined to further reveal the relationship between changes in the TME pH and tumor development (Fig. 2).

### Proinflammatory TME of HCC

Chronic liver inflammation caused by various pathogenic factors is an important basis for the formation of HCC [64]. Immune cells are an important part of the TME, including macrophages, DCs, NK cells, bone marrow-derived suppressor cells (MDSCs), and T/B lymphocytes [65]. The main function of these immune cells is to secrete a large number of inflammatory factors, such as IL-1 and IL-6, to induce an inflammatory response, which also plays a dual role in the regulation of tumor processes [66]. The TME plays an immune monitoring role in the early stage of tumors, mainly through T/B lymphocytes and NK cells, to stimulate anti-tumor immunity and inhibit tumor progression [67]. With the development of the tumor, tumor cells develop immune tolerance under long-term inflammatory stimulation, and excessive inflammation promotes the occurrence and development of the tumor [68]. Together with hypoxia, the upregulation of IL-6 expression by the toll-like receptor signaling pathway in tumor cells promotes the inflammatory response of tumors and promotes tumor resistance, proliferation, and invasion [69]. The initial goal of the inflammatory response is to eliminate foreign invaders or damaged tissue [70]. However, the composition and function of inflammatory cells are often altered in the TME, resulting in immunosuppression and contributing to tumor immune escape [71]. For example, tumor and stromal cells promote inflammation and immunosuppression through NF-κB and STAT3, which promotes tumor cells to evade immune recognition and participate in tumor cell proliferation, metastasis, drug resistance, and tumor angiogenesis [72]. Currently, many methods are being researched and developed to normalize the inflammatory TME in patients with HCC. However, there is no doubt that simultaneously targeting cancer and stromal cells is more effective and more challenging than targeting cancer cells alone (Fig. 2).

### TME promotes immunosuppression in HCC

The TME is a complex internal environment network that tumor cells depend on for survival and development [73]. The immunosuppressive TME is a part of the TME that plays a role in suppressing immune function [74]. Once HCC is formed, the interaction of cellular and non-cellular components in the TME gradually forms an immunosuppressive microenvironment and promotes tumor development [75]. In recent years, the treatment of immune checkpoint blockers (ICBs), such as Nabuliumab (anti-programmed cell death protein 1 [PD-1]) and Attillizumab (anti-programmed cell death ligand-1 [PD-L1]), has revolutionized the treatment landscape of advanced HCC [76]. However, most patients with HCC have primary resistance to immunotherapy and are unable to achieve significant survival benefits [77]. Studies have shown that the key mechanism leading to poor response to ICB therapy lies in the immunosuppressive TME, with MDSCs as the core [78]. The complex interactions between HCC cells and their immunosuppressive microenvironment during HCC development and drug therapy remain to be further studied [79]. Selectively modulating the immunosuppressive regulatory networks associated with primary or secondary tumors may reprogram the microenvironment, providing an immunotherapy strategy for treating HCC [80]. Encouragingly, other molecules with immunosuppressive activity expressed by Tregs, such as T-cell immunoreceptor with Ig and ITIM domains (TIGIT), lymphocyte-activation gene 3 (*LAG3*), and T-cell immunoglobulin and mucin domain-containing protein 3 (TIM3), are also currently in clinical trials [81]. At present, it is urgent to systematically analyze the molecular regulatory mechanisms of the immunosuppressive TME, especially the key pathways of abnormal activation of various inhibitory immune cells, and establish precise combination therapy strategies to break through the bottleneck of the clinical treatment of HCC (Fig. 2).

## Key role of CD73 in HCC progression

**Fig. 3:**
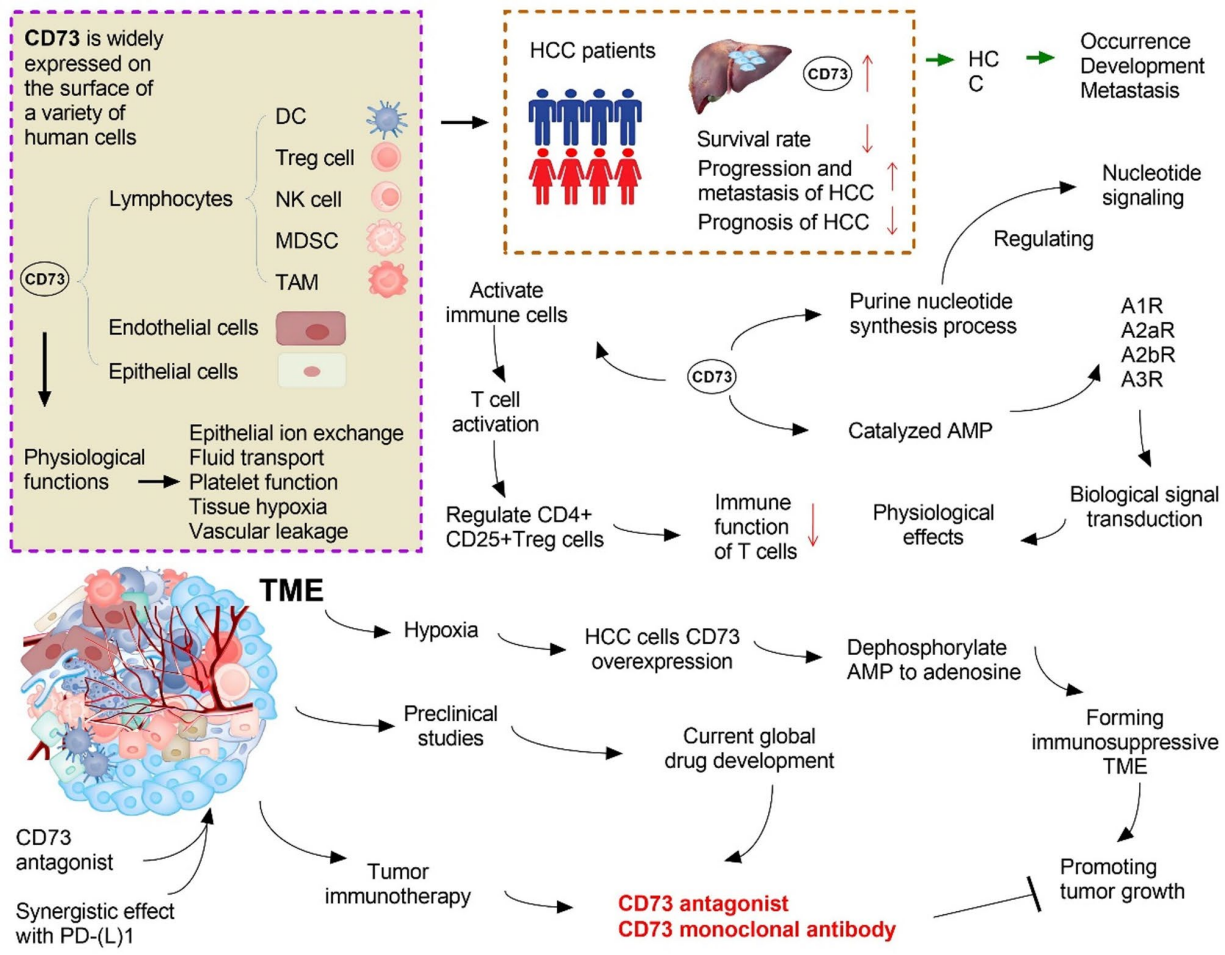
The tumor microenvironment (TME) is an important internal environment in the occurrence and development of tumor diseases. In the TME, CD73 plays an important role in the occurrence, development, and metastasis of hepatocellular carcinoma (HCC) by promoting angiogenesis through immune escape, inflammatory cancer signaling transmission, adhesion molecular function, and neovascularization

The cell types that most frequently express CD73 are various tumor, immune, and stromal cells [82–85]. In solid tumors, especially HCC, CD73 is a key component in the formation of the immunosuppressive microenvironment and the occurrence of tumor immune escape [82]. Human CD73 is a multifunctional transmembrane glycoprotein composed of 523 amino acids encoded by ecto-5’-nucleotidase (*NT5*E) (located at 6q14-21), with a relative molecular mass of 70,000, which is anchored to the cell membrane by glycosylphosphatidylinositol (GPI) [83]. CD73 is widely expressed on the surface of a variety of human cells, including lymphocytes, endothelial cells, and epithelial cells, and controls a variety of physiological functions, including epithelial ion exchange, fluid transport, platelet function, tissue hypoxia, and vascular leakage [84]. CD73 is primarily involved in the following physiological effects: (1) affecting the purine nucleotide synthesis process, where adenosine, mediated by CD73, controls the production of purine nucleotides through CD39 and CD73, thereby regulating nucleotide signaling [85]; (2) catalyzing 5’-adenosine monophosphate (AMP), where the produced adenosine binds to the A1, A2a, A2b, and A3 adenosine receptors, producing different physiological effects through biological signal transduction [86]; and (3) involvement in T cell activation, where adenosine produced by CD73 hydrolysis activates immune cells, affects the proliferation of immune cells, regulates CD4 + CD25 + Treg cells, and reduces the immune function of T cells [87]. CD73, a cell surface enzyme that is widely expressed on the surface of human endothelial cells and lymphocytes (such as Tregs) [88], can convert immune-activating ATP into the immunosuppressant adenosine [89]. Adenosine downregulates immune activity by binding with the downstream adenosine receptor (A2aR) [90]. In the TME, hypoxia induces overexpression of CD73 on the surface of cancer cells to dephosphorylate AMP to adenosine through CD73, thereby forming an immunosuppressive TME and promoting tumor growth [91]. Therefore, CD73 inhibition may activate T cell function. Preclinical studies have shown that CD73 has a good synergistic effect with PD-L1 [92]. Considering the current global clinical development of the CD73 monoclonal antibody, the treatment options explored are mostly a combination of CD73 and PD-1 or other drugs [93]. In the future, CD73 is expected to become a good companion for PD-1 drugs and to further improve the response rate of tumor immunotherapy (Fig. 3).

### CD73-adenosine signaling in the TME mediates immune escape in HCC

**Fig. 4:**
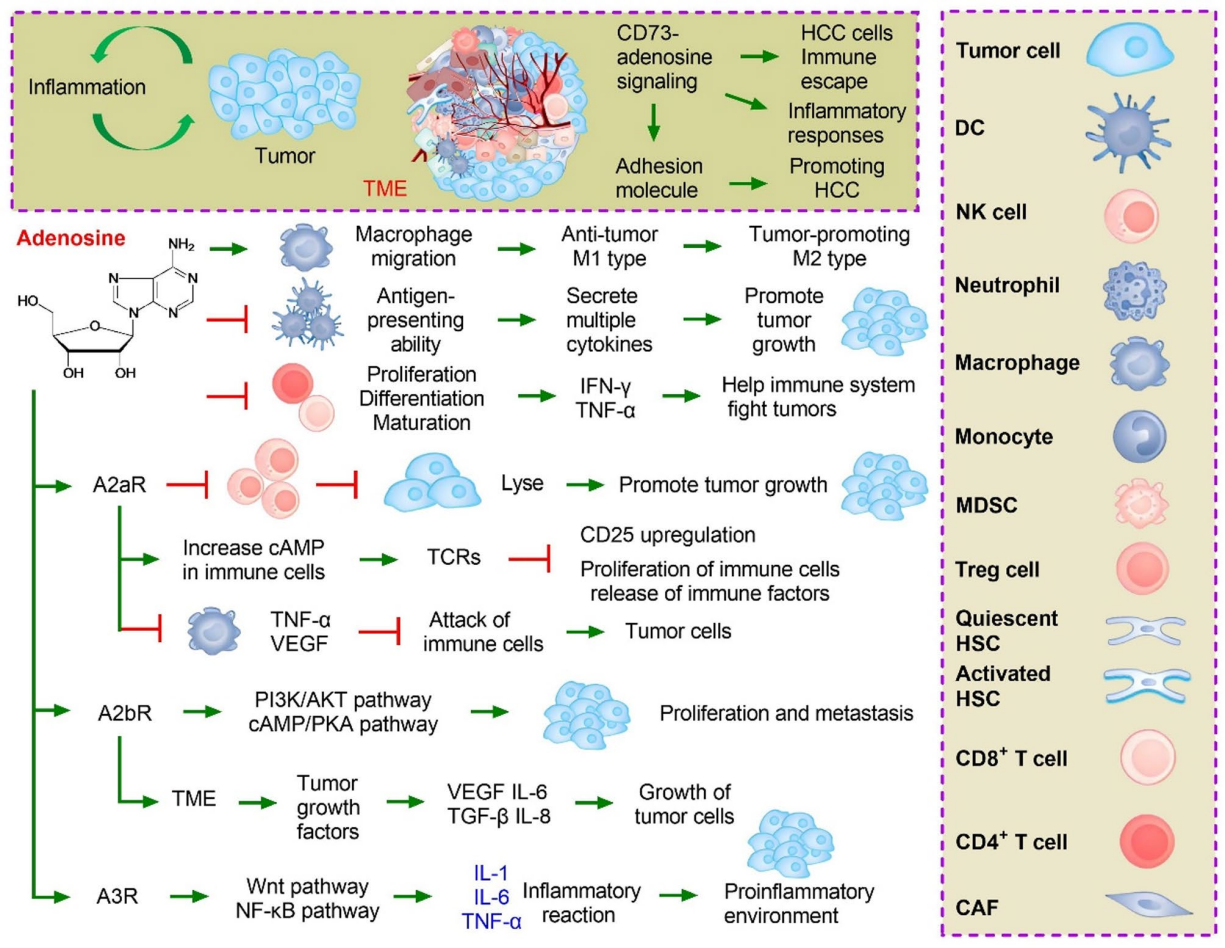
In the tumor microenvironment (TME), CD73 mediates the production and inhibition of inflammatory signaling molecules through the adenosine pathway. CD73 stimulates the production of inflammatory cytokines through various downstream signaling pathways, placing hepatocellular carcinoma (HCC) cells in a homeostasis environment with high expression of inflammatory cytokines, promoting the coexistence of inflammatory cells and cancer cells, and promoting the progression of HCC

Inhibition of CD73 can enhance the anti-HCC immune effect, and the mechanism is related to the inhibition of adenosine accumulation and the reversal of immune system function inhibited by adenosine [94]. The surrounding microenvironment of HCC cells is called the TME and includes various signaling molecules and ECM, including blood vessels in and around the tumor, tumor-infiltrating immune cells, fibroblasts, and PD-1/PD-L1 [95]. HCC can affect its microenvironment through the release of cell signaling molecules, promoting tumor angiogenesis, inducing immune tolerance, and helping tumor cells escape the killing of immune cells within the tumor [96]. The CD73-adenosine pathway is an important pathway involved in tumor immune escape [97]. HCC cells generate immunosuppressive signals mainly by increasing adenosine levels in the microenvironment [98]. Adenosine binds to adenosine receptors in various immune cells to reduce inflammation and inhibit immune responses [99]. First, adenosine can inhibit or affect the maturation, differentiation, and function of various immune cells [100]. For example, adenosine can promote the migration of macrophages from the anti-tumor M1 type to the tumor-promoting M2 type [101]. For DCs, adenosine can inhibit its antigen-presenting ability while inducing DCs to secrete multiple cytokines that promote tumor growth [102]. Second, adenosine can inhibit the maturation of NK cells and significantly decrease their killing activity [103]. For T cells, adenosine inhibits the production of IL-2 by CD4 + T cells, the proliferation, differentiation, and maturation of CD8 + T cells, and the production of a variety of cytokines that help the immune system fight tumors, such as IFN-γ and tumor necrosis factor (TNF)-α. In contrast, adenosine promotes the function of immunosuppressive cells, such as Tregs and MDSCs, to suppress the immune response [104]. Finally, adenosine can increase the expression of immune checkpoints on the surface of immune cells, including PD-1, cytotoxic T lymphocyte-associated antigen 4 (CTLA-4), and LAG3 [105]. Targeted inhibition of CD73-adenosine signaling in the TME has become a promising therapy to alter adenosine levels in the TME and fully restore the anti-cancer function of the immune system. Although drug research targeting the adenosine pathway derived from the TME is promising, it remains in its infancy and its anti-tumor effect still needs to be further confirmed by more large-sample studies (Fig. 4).

### Inhibition of CD73-adenosine signaling reduces inflammatory responses

CD39 upstream of CD73 catalyzes ATP to produce AMP, and the resulting AMP can be converted into adenosine by CD73 [106]. Adenosine binds to downstream adenosine receptors (A2aR or A2bR), which exert a broad immunosuppressive effect by activating the protein kinases protein kinase A (PKA) and c-Src tyrosine kinase (CSK) while inhibiting a series of signaling pathways related to inflammation, such as mitogen-activated protein kinase (MAPK) and protein kinase C (PKC) [107]. In HCC-related inflammation, which is characterized by chronic persistent inflammation, immune cells typically do not exhibit antitumor effects [108]. On the contrary, adenosine can participate in the development, proliferation, and metastasis of HCC by releasing inflammatory mediators and inflammatory-related cytokines into the TME [109]. However, generating an adenosine pathway-induced anti-inflammatory response to enhance the anti-tumor effect and improve the HCC-related inflammatory microenvironment has attracted much attention. Adenosine mediates anti-inflammatory effects through P1 purine receptors (A1R, A2aR, A2bR, A3R) [110]. Adenosine receptors belong to the G protein-coupled receptor family and are expressed by various cells, including immune cells [111]. Adenosine, as a key effector molecule in the regulation of innate and adaptive immunity, can bind to A2aR on the surface of various immune cells to mediate the increase in cAMP in immune cells, thereby inhibiting the upregulation of CD25 mediated by effector T cell receptors (TCRs) as well as inhibiting the proliferation of immune cells and the release of immune factors [112]. Adenosine combined with A2aR can inhibit the production of macrophages, reduce the secretion of different pro-inflammatory mediators such as TNF-α and VEGF, inhibit the attack of immune cells on tumor cells, and help tumor cells to escape immune system control [113]. Adenosine can also affect the differentiation of DCs, mainly through binding with A2bRs, so that it can secrete different tumor growth factors in the immune microenvironment of the tumor, including VEGF, IL-6, IL-8, and TGF-β, providing a good “soil” for the growth of tumor cells [114]. CD73, as one of the main enzyme activators regulating adenosine, plays an important role in the process by which adenosine regulates inflammation and the immune response, responsible for mediating immune escape [115]. In conclusion, CD73 promotes the proliferation and metastasis of HCC cells by mediating adenosine in the process of immune regulation and inflammation. However, the genetic characteristics of CD73/AR and the mechanism underlying the immunosuppressive pathway remain unclear (Fig. 4).

### CD73 in the TME promotes HCC via its adhesion molecule function

CD73 is a glycoprotein that exists on the surface of cell membranes of all cell types and can also be free outside cells [116]. Studies have shown that CD73 in the TME can participate in the proliferation, angiogenesis, and invasion of tumor cells and can also serve as an important adhesion signaling molecule on the cell surface to promote intercellular adhesion, migration, and cancer cell invasion [117]. High expression of CD73 promotes the progression of HCC and is associated with promoting the invasion and metastasis of HCC [118]. The CD73-adenosine signal induces increased inflammation, decreases or loses the adhesion function between normal cells, and leads to cell looseness and disconnection, thereby facilitating invasion and metastasis of HCC cells [119]. CD73 also promotes HCC cell proliferation by regulating the cell cycle, apoptosis, and signaling pathways, such as EGFR, β-catenin/cyclin D1, VEGF, and AKT/extracellular signal-regulated kinase (ERK). Independent of its enzymatic function, CD73 promotes the mutual adhesion, migration, and invasion of HCC cells [120]. New studies have shown that in tumor cells, activation of CD73 can promote the adhesion of tumor cells through the EGFR pathway, produce B-cell lymphoma-2 (Bcl-2) and Bcl-xL to inhibit cell apoptosis, release matrix metalloproteinases (MMPs) to hydrolyze the ECM, and promote the remote migration of tumor cells [121]. In addition, the activation of CD73-adenosine signaling in patients with HCC has been shown to promote inflammation and induce increased levels of E-selectin, intercellular cell adhesion molecule-1 (ICAM-1), vascular cell adhesion molecule-1 (VCAM-1), IL-1, and TNF-α in the blood of patients with HCC [122]. ICAM-1, VCAM-1, and platelet endothelial cell adhesion molecule-1 (PECAM-1) are highly expressed in vascular endothelial cells [123]. Currently, there are few studies on the regulatory role of CD73 in the TME of HCC, and its specific mechanism needs to be further clarified (Fig. 4).

### CD73 in the TME promotes angiogenesis in HCC

**Fig. 5:**
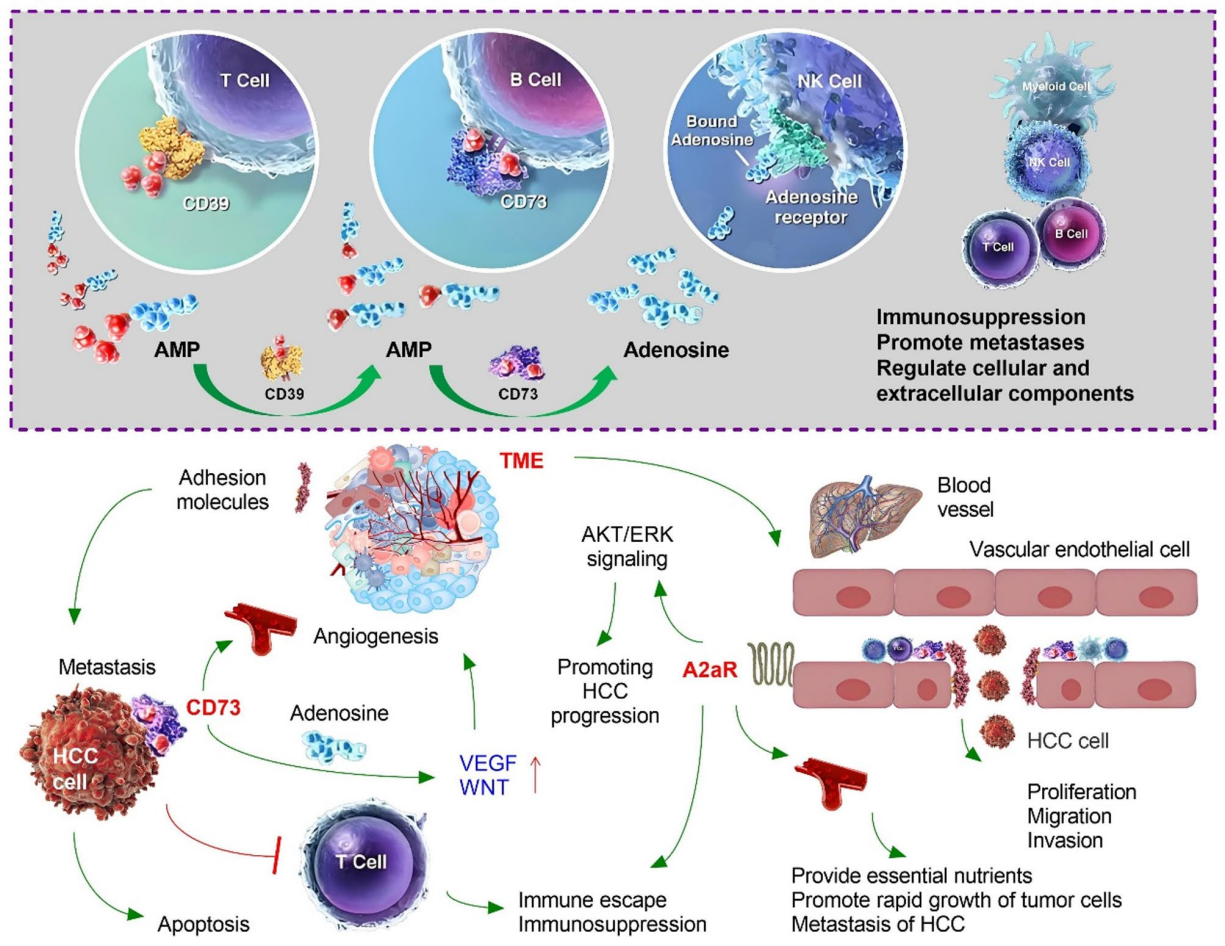
CD73 on tumor cells can effectively promote the formation of tumor blood vessels in the TME, and high expression of CD73 can also promote the proliferation and metastasis of cancer cells. CD73 can stimulate tumor neovascularization through multiple adenosine receptor pathways, as well as protect neovascularization and promote the growth and metastasis of hepatocellular carcinoma (HCC) in vivo

New research has revealed that CD73 plays a key role in promoting tumor angiogenesis. As a rate-limiting enzyme for adenosine synthesis, CD73 drives tumor angiogenesis and promotes rapid tumor growth by promoting the expression of VEGF and HIF-1α [124]. Some studies have found that CD73 + AD-MSCs have higher paracrine activity, which can better promote angiogenesis and have an obvious therapeutic effect on myocardial infarction [125]. CD73 has a non-selective effect on promoting angiogenesis, as well as a strong effect on both normal and cancer tissues. In addition, tumor-associated macrophages (TAMs) in the TME show high expression of CD73, which can promote angiogenesis and lymphogenesis through the secretion of WNT7B, WNT5A, WNT11, VEGF-C, VEGF-D, and other cytokines after activation [126]. TAMs can enhance tumor hypoxia and glycolysis, two important causes of angiogenesis [127]. A new study found that, in the TME, the angiogenic factor VEGF-α mainly exists in T3 neutrophil subsets, and its activation may promote tumor angiogenesis, indicating that T3 neutrophils may be a new target for pathological angiogenesis in tumors [128]. Moreover, HCC cells catalyze AMP to produce adenosine through CD73, which acts on the A2aR of macrophages in the TME and activates its downstream AKT/ERK signaling pathway to induce macrophage proliferation and angiogenesis, thus promoting HCC progression [129]. However, currently, the specific mechanism responsible for reducing HCC angiogenesis by inhibiting CD73 in the TME remains unclear and requires further study. In addition, the possible side effects of the widespread expression of CD73 are issues that remain to be addressed (Fig. 5).

## Different roles of CD73-associated adenosine receptors within the TME of HCC

**Fig. 6:**
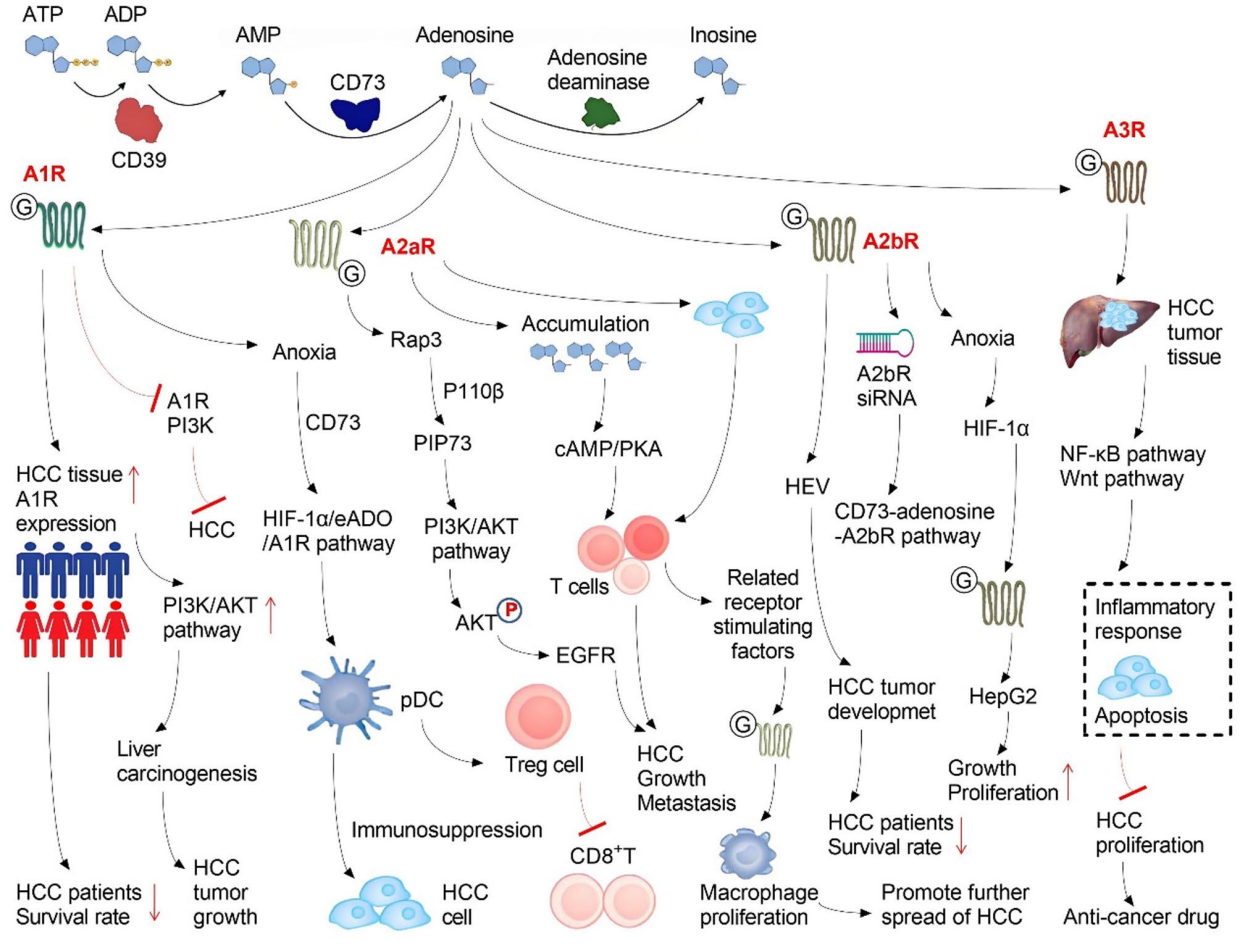
CD73 is expressed in a variety of cells and binds to a variety of adenosine receptors (e.g., A1R, A2aR, A2bR, and A3R) on the surface of hepatocellular carcinoma (HCC) cells by catalyzing the production of adenosine in the tumor microenvironment (TME), thus activating downstream signaling pathways to play a protective role in the disease process and prognosis of HCC

During the development of HCC, the TME interacts with HCC cells to mediate the immune tolerance of HCC, with adenosine playing an important role in this process [130, 131]. CD73 is an ectonucleotidase that works with its upstream signaling molecule, CD39, to convert extracellular ATP into adenosine, which then binds to different adenosine receptors to regulate the immune system and exert anti-HCC effects; however, the associated mechanism has yet to be elucidated [130–135]. Many scholars have conducted research on the regulation of CD73 in HCC cells, especially the CD73 found in the TME of HCC [136–140]. Adenosine mediates anti-inflammatory effects through P1 purine receptors (A1R, A2aR, A2bR, A3R). Adenosine receptors belong to the G protein-coupled receptor family and are expressed by a variety of cells, including immune cells. Adenosine has been found to have a low affinity for the A2bR, and inhibits NK cell maturation via the A2aR in the innate immune system [132]. The differentiation of monocytes into macrophages has been shown to be inhibited by the A2bR, which also promotes macrophages from the pro-inflammatory M1 type to the anti-inflammatory M2 type [133]. Similarly, Adenosine acts on DCs through the A2bR, allowing them to secrete vasotropic and immunosuppressive factors that further promote tumor cell growth [134]. In the acquired immune response system, Adenosine inhibits the production of IL-17 by CD4 + T cells through A2aR and A2bR, and promotes the transformation of CD4 + T cells into immunosuppressive cells [135]. At high concentrations, adenosine has been shown to inhibit the proliferation, differentiation, maturation, and cytokine production of B cells via A2aR and A3R [136]. Adenosine also inhibits the proliferation, differentiation, and maturation of CD8 + T cells, as well as the production of cytokines such as IL-2, INF-γ, granulocyte-macrophage colony-stimulating factor (GM-CSF), and granzyme B through A2aR and A3R [137]. On the contrary, adenosine promotes the function of immunosuppressive cells, such as CD4 + Tregs and MDSCs, through A2aR or A2bR [138]. In general, adenosine inhibits the functions of NK cells, DCs, and cytotoxic T lymphocytes (CTLs) through its receptors, while enhancing the activity of immunosuppressive cells and participating in the formation of TME immune tolerance [139]. At the same time, adenosine acts on vascular endothelial cells and stromal cells, promoting the formation of TME neovascularization and providing the necessary conditions for tumor metastasis [140]. In summary, the following strategies can be adopted to block the role of adenosine in the TME: (1) preventing the synthesis of adenosine; (2) preventing adenosine from binding to its receptor; and (3) preventing the degradation of high concentrations of extracellular adenosine (Fig. 6).

### A1 adenosine receptor in HCC

The hypoxic TME of HCC facilitates plasmacytoid dendritic cell (pDC) recruitment, the mechanism of which is related to activation of the HIF-1α/adenosine/A1R signaling pathway [141]. Tumor-derived adenosine promotes the immunosuppressive phenotype of pDCs, leading to the depletion of cytotoxic CD8 + T cells and the amplification of Tregs [142]. Notably, in an immunoactive HCC mouse model, monoclonal antibodies or A1R inhibitors have been shown to significantly inhibit HCC growth by enhancing tumor-killing immunity [143]. Therefore, targeted inhibition of A1R to downregulate pDC recruitment may serve as a potential adjunct strategy for HCC immunotherapy [144]. The immunosuppressive TME and unsatisfactory T-cell persistence are the main causes of nutrient competition problems in the immune microenvironment. For example, researchers have engineered chimeric antigen receptor T (CAR-T) cells to express membrane-bound CD26 and cytoplasmic A1R, converting adenosine to inosine. They found that A1R, in turn, activated CAR-T cells in the autocrine response to CD3/CD26 stimulation, improving migration and resisting TGF-β1 inhibition. The fusion of ADA1 with anti-CD3 single chain antibody fragment (scFv) further promoted the production of inosine and reduced tumor cell uptake. In a mouse model of HCC, these researchers found that metabolically supplemented CAR-T cells showed improved tumor-reduction ability; this new approach has the potential to provide selective inosine supplementation to enhance the efficacy of CAR-T therapy for HCC [145]. In addition, AR1 can trigger the signal transduction of the (phosphatidylinositol-3-kinase) PI3K/AKT/glycogen synthase kinase (GSK)-3β/β-catenin intracellular carcinogenic pathway [146]. The A1R agonist 2-Chloro-N6-cyclopentyladenosine (CCPA) enhances ischemia-reperfusion (IR) damage, intracellular steatosis, and oxidative species (OS) production, thereby further increasing lipid/OS-dependent apoptosis signal regulating kinase 1 (ASK1)-c-jun N-terminal kinase (JNK) stimulation [147]. In conclusion, several studies have found that A1R deletion inhibits the growth of various tumor cell lines and tumor development in immunodeficient xenografts both in vitro and in vivo [148–150]. Therefore, A1R represents a promising research area (Fig. 6).

### A2a adenosine receptor in HCC

The inhibition of A2aR is a promising cancer immunotherapy approach that is currently being evaluated in several clinical trials [151–153]. In patients with HCC, the RNA expression of *A2aR* in the tumor tissues has been shown to be higher than that in other tumor tissues [154, 155]. Adenosine signaling is regulated by the anoxic immune microenvironment in the liver. After treatment with the A2aR inhibitor SCH58261 combined with anti-PD1, peripheral blood A2aR + T cells showed high proliferation in patients with HCC. In an in situ mouse model of HCC, the combination of SCH58261 and anti-PD1 activated T cells and reduced tumor size. This suggests that A2aR blocking promotes an immunotherapy effect in mouse HCC models, highlighting the clinical benefit in patients with advanced HCC [154, 155]. Mechanistically, adenosine produced by CD73 binds to the adenosine A2aR and activates Rap1, which recruits P110β to the plasma membrane and triggers the production of phosphatidylinositol 3-phosphate (PIP3), thereby promoting the phosphorylation of AKT in HCC cells [156]. Recent studies have found that both mouse and human double-negative T (DNT) regulatory cells overexpress CD39, protect DNT from extracellular ATP-induced apoptosis, and produce adenosine together with CD73, thereby inducing high levels of neutrophil apoptosis [157]. Notably, in human HCC, low expression of A2aR is associated with cirrhosis, liver inflammation, and poor survival, suggesting the cautious use of A2aR antagonists in patients with HCC [158]. Current research is focused on the screening of novel A2aR antagonist candidates with strong A2aR antagonist activity, good hepatic microsomal metabolic stability, and good oral bioavailability for cancer immunotherapy (Fig. 6) [159, 160].

### A2bR adenosine receptor in HCC

Adenosine is a metabolite that suppresses the anti-tumor immune response of T and NK cells via extracellular binding to two subtypes of A2Rs [161, 162]. CD39 + T-cell infiltration and adenosine receptor A2bR expression levels in the tumor TME have been shown to be negatively correlated with overall survival in patients with HCC [163]. Three-dimensional morphological analysis of the mitochondrial network revealed that these ARs are potential regulators of mitochondrial energy metabolism due to increased ATP production and coupling efficiency in the presence of BAY 60-6583 (A2bR agonist) [164]. Intracellular ATP is released into the extracellular space and degraded into adenosine, which acts as a signaling molecule to activate the cyclic adenosine phosphate (cAMP) pathway through the A2bR, thereby promoting the polarization of M2 macrophages [165]. The A2bR antagonist ISAM-R56A, can promote the proliferation of T and NK cells, the production of IFN-γ and perforin, and increase the infiltration of tumor-infiltrating lymphocytes into tumor spheres, without altering the expression of adhesion molecules. The A2bR is a promising target in immunotherapy, with A2bR and A2aR/A2bR double antagonists showing similar or better results than the A2aR antagonist AZD-4635 [166]. The expression of CAF-CD73 is enhanced by A2bR-mediated feedforward circuitry triggered by tumor cell death, thereby reinforcing the CD73 checkpoint [167]. The activation of A2bR and the induction of NADPH oxidase 2 (NOX2)-dependent oxidative stress within endothelial cells promote the interaction between endothelial and stromal cells, thereby promoting tumor angiogenesis [168]. The human A2bR is a G protein-coupled receptor of class A, which has a relatively low affinity for its ligand adenosine and the A2bR agonist NECA. The two structures of A2bR bind NECA and BAY60-6583. The residues V2506.51 and N2737.36 are the key determinants of its selectivity to A2bR [169]. Single A2bR antagonists and A2aR/A2bR double antagonists with good liver microsomal stability are future research directions for HCC immunotherapy (Fig. 6) [170].

### A3R adenosine receptor in HCC

A3R is overexpressed in human HCC cells [171]. Namodenoson (CF102) is an A3R agonist that induces dysregulation of the Wnt/β-catenin and NF-κB signaling pathways leading to apoptosis in HCC cells and is currently being used in phase III trials in advanced HCC [172, 173]. Additionally, the specific A3R agonist MECA, which has antitumor effects through both A3R-dependent and -independent pathways, has shown antiproliferative effects against various tumor types, especially HCC [174]. Approaches from the opposite direction also have potential, such as the new dual A2aR/A3R nucleoside antagonists that are currently under development, which represent promising candidates for immunooncology [175]. Recent findings indicate that inhibition of A3R by a highly selective antagonist of A3R, FM101, or gene deletion alleviates inflammation and fibrosis in metabolic dysfunction-associated steatotic liver disease (MASLD) by inducing mitochondrial dysfunction and subsequent necrosis of proinflammatory monocyte-derived KCs (MoKCs) [176]. In addition, the A3R agonist Namodenoson improves liver function/pathology in patients with non-alcoholic fatty liver disease [177]. A3R has become a target for drug development, and the identification of potent and highly selective A3R agonists is the current research direction (Fig. 6) [178–181].

## Anti-HCC agents targeting CD73-adenosine signaling

The overexpression of CD73 and the four types of adenosine receptors in a variety of tumor cells is closely related to the regulation of the TME, inhibition of the tumor immune response, tumor metastasis, drug resistance, and patient prognosis, which also provides a basis for the development of drugs targeting CD73 and adenosine receptors in clinical therapy. At present, many pharmaceutical companies worldwide are actively developing adenosine pathway drug research and development, which is still in the clinical stage. Currently, there are no approved drugs on the market, and the fastest progress of drug development for clinical phase II is currently underway. From the current clinical trials, drug combination represents the most important direction for CD73-adenosine pathway drug development.

### Anti-HCC drug candidates targeting CD73

At present, there are 18 monoclonal antibody drug candidates targeting CD73 in clinical stages worldwide, 17 of which are related to the regulation of the immune microenvironment (Table 1). The bispecial antibody targeting CD73 is represented by AK131 of Akesobio, which is also the only CD73 bispecial antibody to have entered the clinical stage worldwide. AK131 has shown strong activity in vivo and in vitro, not only effectively blocking the PD-1/PD-L1 interaction but also effectively promoting the activation of T and B cells and inducing the endocytosis of CD73 (Table 2). Currently, there are a total of five small molecule drug candidates targeting CD73 entering the clinical stage, four of which are related to HCC, and Quemliclustat is the fastest developing drug worldwide. A small-molecule inhibitor targeting CD73 inhibits AMP and extracellular adenosine-mediated tumor immunosuppression by effectively blocking adenosine production in the TME. The drug candidate was initiated in a phase III trial on September 23, 2024 (PRISM-1/NCT06608927), becoming the first small molecule CD73 inhibitor to enter phase III (Table 3). Currently, two drug candidates of the CD73-antibody-drug conjugate (CD73 ADC) are entering the clinic, both of which are in the first clinical phase. Studies have shown that, compared to uncoupled CD73 naked antibody, CD73 ADCs can induce the accumulation of pro-inflammatory macrophages and activated DCs in tumors. At the same time, CD73 ADCs also have the function of protecting effector T cells and stimulating DCs, which has the dual advantages of killing CD73-highly expressed tumors and improving the tumor immune response. However, the specific effect requires further clinical verification (Table 4).

**Table 1:** Advances in monoclonal antibodies targeting CD73

Drug Candidate	Company	phase	Indication	Type
Oleclumab	AstroZeneca,	III	Advanced malignant solid tumor	Antibody
	Medlmmune			
Uliledlimab	I-mab Biopharma Halozyme	II/III	Advanced malignant solid tumor, Gastrointestinal tumor	Antibody
BMS-086179	Therapeutics	II	Advanced malignant solid tumor	Antibody
	Jacobio		Solid tumor, Advanced cancer, Advanced malignant solid tumor	
JAB-BX102	Pharmaceuticals	II	Advanced malignant solid tumor	Antibody
	Akesobio		Advanced malignant solid tumor,	
Dresbuxelimab	Huabo Biopharm	I/II		Antibody
HB0045	Huaota Biopharm	I/II	Solid tumor	Antibody
Sym-024	Symphogen A/S,	I/II	Advanced malignant solid tumor,	Antibody
	Servier Group		Solid tumor, Tumor metastasis	
Ansipastobart	Bioraypharm	I	Advanced malignant solid tumor, Solid tumor	Antibody
Anti-CD73-mAb	BMS	I	Cancer	Antibody
HBM-1007	Harbour BioMed	I	Cancer	Antibody
IBI-325	Innovent	I	Advanced malignant solid tumor, Advanced cancer	Antibody
IPH5301	Innate Pharma	I	Advanced malignant solid tumor	Antibody
Mupadolimab	Corvus	I	Advanced malignant solid tumor	Antibody
	Pharmaceuticals			
PM-1015	Biotheus	I	Advanced malignant solid tumor, Advanced cancer	Antibody
PT-199	Phanes	I	Advanced malignant solid tumor, Advanced cancer	Antibody
	Therapeutics			
Uprevstobart	Incyte Corp	I	Advanced malignant solid tumor	Antibody
Recombinant anti-CD73 mAb	Henlix Biotech	Apply	Solid tumor	Antibody

**Table 2:** Bispecific antibody (BsAb) targeting CD73

Candidate	Target	Mechanism	Indication	Company	Phrase
AK-131	CD73×PD-1	CD73 antagonist, PD-1 antagonist, ADCC, T lymphocyte stimulant	Advanced malignant solid tumor, Autoimmune disease	AKEsobio	I
AK-137	CD73×LAG3	CD73 conditioning agent, LAG3 conditioning agent	Advanced cancer	AKEsobio	Apply
HB-0046	CD39×CD73	CD39 antagonist, CD73 antagonist	Advanced malignant solid tumor	Huaota Biopharm	Apply

**Table 3:** Small molecule drug candidates targeting CD73

Candidate	Target	Mechanism	Indication	Company	Phrase
Quemliclustat	CD73	Blocking adenosine production in the TME inhibits AMP and extracellular adenosine-mediated tumor immunosuppression	Advanced bile duct cancer, cancer	Arcus, Gilead, Sarah Cannon	III
ABSK-051	CD73	Blocking adenosine production in the TME	Advanced malignant solid tumor, cancer	Abbisko Therapeutics	I
CB-708	CD73	Blocking adenosine production in the TME	Locally advanced/metastatic solid tumor	Antengene, Calithera	I
BPI-472,372	CD73	Blocking adenosine production in the TME	Advanced malignant solid tumor	Betta pharma	Apply

**Table 4:** Drug candidates of antibody-drug conjugates (ADCs) targeting CD73

Candidate	Target	Mechanism	Indication	Company	Phrase
BB-1709	CD73	Recruit and aggregate macrophages and activated DCs in the tumor	Solid tumor, solid tumor expressing CD73	Bliss Biopharm	I
HB-0052	CD73	Recruit and aggregate macrophages and activated DCs in the tumor	Advanced malignant solid tumor, solid tumor	Huaota Biopharm	I

### Anti-HCC drug candidates targeting adenosine and its receptors

Adenosine overproduction occurs at all stages of tumorigenesis, making the adenosine pathway an attractive but challenging therapeutic target. Currently, much of the research in immuno-oncology is focused on restoring immune surveillance, primarily by blocking adenosine-producing enzymes in the TME and adenosine receptors on immune cells through single or combination drugs. The number of clinical trials targeting adenosine pathway components has increased in recent years; however, most trials are still in the early stages of development, with a few being in the clinical stage; additionally, no drugs are approved for marketing, and the most advanced drugs under development have just entered clinical phase II (i.e., NCT02655822, NCT04280328, NCT03207867, NCT04895748, NCT03549000, NCT03381274, NCT04089553, NCT02403193, NCT03720678, NCT03629756, NCT04262856, NCT04892875, NCT05060432, NCT04233060, and NCT03873883) (Table 5).

**Table 5:** Immune therapeutic drug candidates targeting the adenosine pathway

Candidate	Target	Indication	Company	Phrase
CPI-444	A2aR	Combination with a PD-L1 monoclonal antibody and/or CD73 monoclonal antibody for treatment of advanced solid tumors	Roche/Corvus	I/II
PBF-509	A2aR	Combination with a PD-L1 monoclonal antibody and/or CD73 monoclonal antibody for treatment of advanced solid tumors	Novartis	I/II
CS3005	A2aR	Advanced solid tumors	Cstone Pharma	I
SHR5126	A2aR	Advanced solid tumors	Hengrui Pharma	I

## Treatment and prospects

Overactivation of CD73-adenosine pathways in the TME induces immunosuppressive signals and promotes the development and progression of HCC. The proliferation and metastasis of tumors can be promoted through an immunosuppressive pathway, a mechanism that prevents the immune system from eliminating malignant tumor cells and allows the disease to progress from an early stage to a deadly state. Since the discovery of tumor immunotherapy targeting the “immune-escape” mechanism, great breakthroughs have been made in cancer treatment. Immune checkpoint inhibitors (e.g., PD-1/PD-L1, CTLA-4) have shown excellent therapeutic effects on various tumors. Large- and small-molecule drugs targeting immune checkpoints have also become a research hotspot. Despite these successes, there remain some problems to be solved, such as the limited indications and immune resistance in some patients.

In the CD73-adenosine pathway, CD39, CD73, and A2aR are the three key molecules for the production and function of adenosine. As a biomarker of tumors, the expression level of adenosine pathway-related molecules is significantly correlated with the clinicopathological features and prognosis of tumors. In addition, the adenosine pathway has broad application prospects in the evaluation of immunotherapy effects. A variety of targeted inhibitors have been developed, including CD39 inhibitors, CD73 inhibitors, and A2aR blockers, many of which have shown exciting clinical activity, but also have low selectivity, metabolic instability, low water solubility, or high plasma protein binding degree shortcomings or deficiencies. However, drug research targeting the CD73-adenosine pathway has just begun, and its anti-HCC effect needs to be further confirmed by more large-scale studies. Given that adenosine production depends on the hypoxic environment and cell renewal, in the future, CD73-adenosine pathway inhibitors may be combined with anti-HCC agents that induce hypoxia and cell death. Synergies with other immunotherapies, such as the combination of PD-1, PD-L1, and CTLA-4 antibodies, can also be explored. In conclusion, the CD73-adenosine pathway plays an important role in tumor immunity, and the CD73-adenosine pathway will show great clinical translational potential with the continuous development of research.

The CD73-adenosine pathway is a new target for tumor immunotherapy due to immune system suppression caused by adenosine production in the TME. Through drug screening for molecular targets in the CD73-adenosine pathway, lead compounds, monoclonal antibodies, and small-molecule CD73 inhibitors have been obtained. Blocking antibodies and small molecule inhibitors targeting the CD73-adenosine pathway have shown good anti-tumor efficacy; however, poor metabolic stability, low inhibition efficiency, low selectivity, high potential for drug resistance, and binding site mutations leading to competitive inhibitor resistance, are still the main problems in drug development. CD73 is the main immunosuppressive mediator of the TME, which acts primarily through the production of extracellular adenosine. In addition to the inherent effects of CD73 in tumor cells on tumor cell proliferation, angiogenesis, invasion, and metastasis, the expression of CD73 by tumor and immune cells also weakens anti-tumor immunity by inhibiting the function of protective immune cells (e.g., effector T cells, NK cells, DCs, and B cells) while maintaining the function of regulatory immune cells (e.g., Tregs, MDSCs, TAMs, and CAFs). Improving the response rate of immunotherapy and increasing the efficacy of immunotherapy for treating tumors are the focus of many researchers at present.

## Data Availability

No datasets were generated or analysed during the current study.
